# Supplementary figures and images for: Beyond the mother-child dyad: Is co-residence with a grandmother associated with adolescent girls’ family planning knowledge?
Source: PLoS One. 2022 Mar 15;17(3):e0265276. doi: 10.1371/journal.pone.0265276 (PMC8923440; doi:10.1371/journal.pone.0265276)

# S1 Appendix


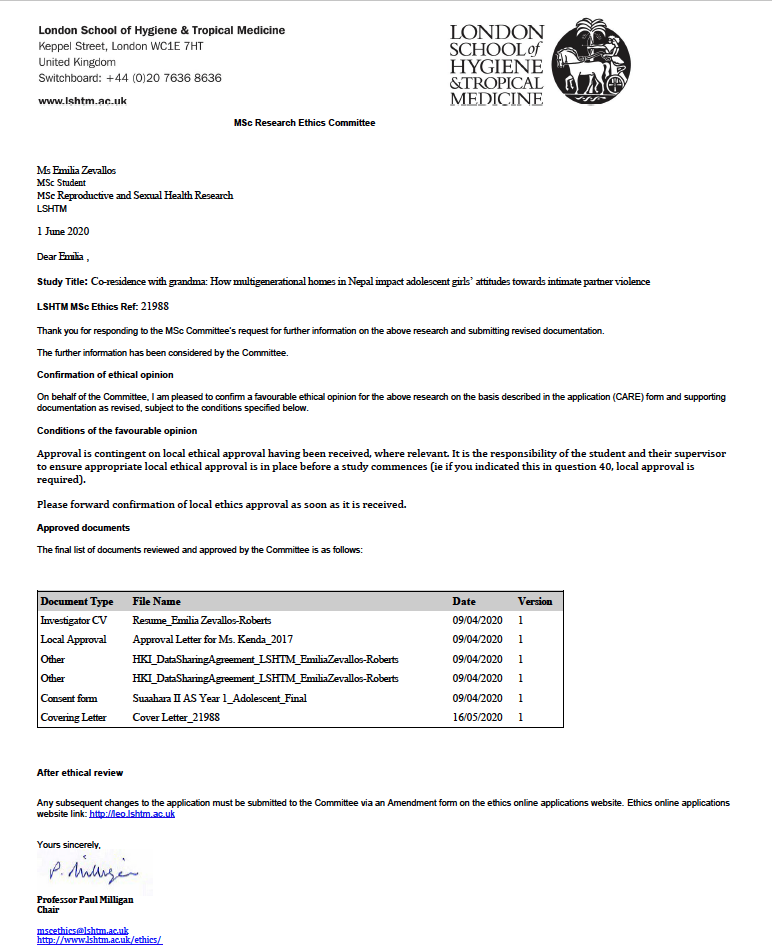

Supplement: S1 Appendix — (DOCX) [file pone.0265276.s001.docx]
